# Importance of Boreal Rivers in Providing Iron to Marine Waters

**DOI:** 10.1371/journal.pone.0107500

**Published:** 2014-09-18

**Authors:** Emma S. Kritzberg, Ana Bedmar Villanueva, Marco Jung, Heather E. Reader

**Affiliations:** Department of Biology/Aquatic Ecology, Lund University, Lund, Sweden; Auckland University of Technology, New Zealand

## Abstract

This study reports increasing iron concentrations in rivers draining into the Baltic Sea. Given the decisive role of iron to the structure and biogeochemical function of aquatic ecosystems, this trend is likely one with far reaching consequences to the receiving system. What those consequences may be depends on the fate of the iron in estuarine mixing. We here assess the stability of riverine iron by mixing water from seven boreal rivers with artificial sea salts. The results show a gradual loss of iron from suspension with increasing salinity. However, the capacity of the different river waters to maintain iron in suspension varied greatly, i.e. between 1 and 54% of iron was in suspension at a salinity of 30. The variability was best explained by iron:organic carbon ratios in the riverine waters – the lower the ratio the more iron remained in suspension. Water with an initially low iron:organic carbon ratio could keep even higher than ambient concentrations of Fe in suspension across the salinity gradient, as shown in experiments with iron amendments. Moreover, there was a positive relationship between the molecular size of the riverine organic matter and the amount of iron in suspension. In all, the results point towards a remarkably high transport capacity of iron from boreal rivers, suggesting that increasing concentrations of iron in river mouths may result in higher concentrations of potentially bioavailable iron in the marine system.

## Introduction

In limnic, estuarine as well as marine waters, iron (Fe) is of profound importance to the mobility, bioavailability and biogeochemistry of many elements and compounds [1]. Iron is also essential as a micronutrient to all organisms, and a co-factor in many enzymes that control key processes such as photosynthesis and cellular respiration. While Fe is one of the most abundant elements in soils and sediments, its concentration in marine waters is often very low (e.g., 0.01–0.8 nmol L^−1^ in ocean waters [2];). This scarcity of Fe in marine waters is the result of the low solubility of Fe(III), which is the predominant oxidation state in oxygenated surface waters in the neutral pH range [1]. In freshwaters, interactions with organic matter (OM) maintain Fe in suspension where it would otherwise precipitate and sediment [3]. Although OM is also enhancing Fe solubility in marine waters [4], the ability of the OM to maintain Fe in suspension decreases quickly as salinity increases [5], [6]. Thus while Fe concentrations in limnic systems are relatively high and rarely limiting (but see [7]), the nano and subnanomolar concentrations in marine waters mean it is a limiting factor of primary production in half of the global ocean [8]–[10], e.g., the Fe limitation of the so-called High Nutrient Low Chlorophyll regions [11].

Recent research has shown that Fe concentrations in Swedish and Finnish rivers draining into the Baltic Sea have increased over the last decades [12], [13]. These rivers encompass a broad climate and vegetation gradient and a striking trend was seen across the entire gradient. In the Swedish systems Fe concentrations have on average doubled over four decades (1972–2010), but there is a large variation among the rivers with increases between 20 and 470%, or 1 and 28 µmol L^−1^ [12]. In the Finnish rivers studied by Sarkkola et al. [13] Fe concentrations increased on average by 40% from 1995 to 2006. Neal et al. [14] report similar trends in the Upper River Severn catchment of mid-Wales, with a doubling of Fe concentrations over 20 years. Along with these positive trends for Fe concentrations follows increasing coastal export. From catchments in southern Sweden, which are dominated by boreal forests, Fe export has increased by 400% [12]. Given the decisive role of Fe to a number of ecosystem functions, this is a phenomenon that may have far reaching implications for the aquatic ecosystems.

The consequences that this marked and continuous increase in Fe export may have for the receiving ecosystem depend on the fate of the Fe along the salinity gradient. Fe is known to behave non-conservatively [5], [6], i.e. it has been estimated that at least 95% of the Fe aggregates and sediments in the estuaries [15], and consequently riverine waters traditionally were not considered an important source of Fe to marine waters. However, the ability of the OM to maintain Fe in solution appears to vary with the nature of OM [16]–[19]. For example, fulvic acids (FA) have been suggested to have a high potential to carry trace metals in saline waters [16], [17], [20]. Krachler et al. [16], [17] found that as much as 22% of Fe from peat draining waters with high FA concentrations remained in solution at high salinity and that this water could provide 3.3 µmol L^−1^ soluble Fe to marine waters. This is in stark contrast to e.g. a 95% loss of Fe during estuarine mixing and ∼40 nmol L^−1^ Fe provided by the Ob and Yenisey rivers [21].

The aim of this study was to assess the stability of Fe from seven Swedish rivers draining into the Baltic Sea, and to understand which factors may determine variation in the stability of Fe over salinity gradients. This is a question of particular relevance for the Baltic system, where on the one hand relatively high Fe concentrations have been suggested as one factor contributing to the dominance of cyanobacteria during summer blooms [22], [23], while on the other hand several studies report that Fe may periodically act as a limiting nutrient [24]–[27]. The rivers studied here have catchments dominated by boreal forests, and the rivers exhibit high and increasing Fe concentrations. Whether the increasing coastal Fe export through these rivers translates into higher Fe concentrations in the receiving water depends on the transport of the Fe through the salinity gradient.

## Materials and Methods

### Study sites and sample collection

Seven rivers in the south of Sweden, draining in to the Baltic proper (Emån, Ljungbyån, Lyckebyån, Mörrumsån and Helgeån) and Kattegat (Lagan and Nissan), were included in this study (Table 1). Temporal trends in Fe and OM concentration (measured as chemical oxygen demand) were analyzed based on data from 1972 until 2012 from the Swedish national monitoring program run by the Swedish University of Agricultural Sciences. Samples were taken monthly around the middle of the month, meaning that the trend analyses are based on ∼490 data points for each river and include seasonal variability. Sampling and analyses were performed according to standard limnological methods, using national/international standards and subjected to quality control routines as defined in the accreditation of the SWEDAC accredited laboratory. A detailed description of methods as well as data is freely available at http://webstar.vatten.slu.se/db.html. Chemical oxygen demand was assessed as consumption of KMnO_4_ in unfiltered water. There was a change of titrator model in 2008. Iron was determined on unfiltered samples preserved with 0.5% HNO_3_ by atomic adsorption spectroscopy until 1993 and inductively coupled plasma atomic emission spectroscopy (ICP-AES) from 1994 and onwards. Previous examination of these data series for abrupt shifts suggest that changes in analytical methods do not affect temporal trends [12]. Sampling for experiments was performed from June 2012 until January 2013. No specific permissions were required for this sampling, which did not involve endangered or protected species. Two rivers (Emån and Helgeån) were sampled twice to include a degree of temporal variability. The rivers were sampled by taking grab samples with acid washed 5-liter polyethylene carboys. Since riverine colloids are easily destabilized, the samples were immediately returned to the laboratory and filtered through pre-combusted (450°C>4 hours) Whatman GF/F filters (nominal pore size 0.7 µm) and the experiments started as soon as possible. The filtered waters were stored in darkness at 4°C up to four days. All the equipment used for sampling as well as experimental work was acid washed (10% HCl) and rinsed with deionized water (milli-Q water) before use.

**Table 1:** Changes in median iron and organic matter concentrations in the rivers since the beginning of monitoring until 2012.

Sampling site		Iron			Organic matter		
		1972–1976	2008–2012	Increase	1972–1976	2008–2012	Increase
		(µmol L^−1^)	(µmol L^−1^)	(µmol L^−1^) (%)	(mmol O_2_ L^−1^)	(mmol O_2_ L^−1^)	(mmol O_2_ L^−1^) (%)
Emån	57°08′28′′N, 16°27′09′′E	7 (6–9)	14 (11–16)	7 (99)	0.43 (0.37–0.53)	0.48 (0.42–0.57)	0.06 (13)
Ljungbyån	56°37′53′′N, 16°10′21′′E	3 (1–5)	18 (14–23)	15 (523)	0.24 (0.19–0.35)	0.51 (0.41–0.64)	0.27 (113)
Lyckebyån	56°11′55′′N, 15°39′47′′E	12 (7–17)	32 (28–38)	20 (175)	0.28 (0.24–0.33)	0.66 (0.55–0.80)	0.37 (131)
Mörrumsån	56°11′21′′N, 14°45′00′′E	2 (1–3)	10 (8–12)	8 (444)	0.27 (0.25–0.30)	0.47 (0.43–0.55)	0.21 (76)
Helgeån	55°56′36′′N, 14°13′09′′E	9 (5–15)	34 (29–45)	25 (284)	0.30 (0.25–0.35)	0.66 (0.58–0.74)	0.36 (118)
Lagan	56°30′56′′N, 13°03′05′′E	7 (6–7)	18 (15–21)	11 (170)	0.31 (0.27–0.35)	0.51 (0.48–0.58)	0.20 (65)
Nissan	56°41′29′′N, 12°52′25′′E	10 (8–11)	21 (17–27)	12 (120)	0.36 (0.32–0.43)	0.58 (0.48–0.72)	0.22 (61)

The first and third quartiles are given within brackets.

### Aggregation experiments

Experiments were designed to test the effect of salinity, Fe concentration and OM properties on the aggregation of riverine dissolved Fe. To study the effect of salinity, an artificial salt mixture (38.08 g NaCl, 7.25 g MgCl_2_, 5.8 g Na_2_SO_4_, 0.96 g KCl, 1.6 g CaCl_2_ and 0.29 g NaHCO_3_, following [28]) dissolved in deionized water was added to filtered river water. In 50-ml Falcon tubes 15 ml of salt mixture of different concentrations was added to 35 ml of river water to create a salinity gradient from 0 to 30 in 8 to 12 steps. The samples were gently mixed and left to settle at 15°C. After 24 hours, salinity and pH was measured and the samples were centrifuged for 8 hours at 4500 rpm at room temperature. Following centrifugation a pipette was used to withdraw samples from the supernatant for Fe (3 mL) and OM (20 mL) analyses. The standardized experimental temperature meant that any effect of temperature on Fe solubility was excluded. Previous studies suggest that temperature has a minor effect on Fe solubility in coastal waters [29], probably due to interactions with organic matter. Moreover, solubility is generally higher at lower temperatures [29]–[31] and since 15°C is in the upper range of temperatures measured in rivers, estuaries and open waters of this region, presented numbers of Fe in suspension in this study should represent conservative estimates.

The effect of increasing Fe concentration on the aggregation process was studied on a subset of the river waters (Emån, Lyckebyån and Helgeån). Data from the Swedish national monitoring program from 1972 was used to fit a linear regression to extrapolate the increase in Fe concentration from 2012 to 2080. Different volumes (20–636 µL) of 0.03 M FeCl_3_ in a solution of 0.001 M HCl were added to 35 mL of river water, resulting in elevated Fe concentration by 53, 52 and 76% in Emån, Lyckebyån and Helgeån, respectively. The FeCl_3_ addition caused a drop in pH due to the acidity of the solution and pH was immediately readjusted to *in situ* level using 0.1 M NaOH. After one hour pH was measured again to check and adjust for any drift. To produce the salinity gradient the same procedure as described above was followed.

The addition of salt elevated the pH of the river samples. To be able to separate the effect of salinity and pH on the aggregation process, an experiment where only pH was manipulated was done with water from Emån and Lyckebyån. pH was adjusted by addition of 0.1 M NaOH and HCl aiming to produce a pH gradient between 6.5 and 8.5 in increments of 0.5 and Fe aggregation was estimated as above.

### Analytical methods

Salinity was determined with a WTW inoLab conductimeter and pH was measured using a Mettler Toledo SevenGo pH-meter.

Fe was determined with the ferrozine method, which provides robust measurements of Fe at the submicromolar level [32], [33]. Addition of hydroxylamine hydrochloride reduced Fe(III) in the samples to Fe(II), so that Fe(II) analyzed corresponded to total Fe (i.e. Fe(II) + Fe(III) of the sample) [32], [33]. The preparation of reagents was made according to [33]. The sample water (2.5 mL), ferrozine (250 µL) and hydroxylamine hydrochloride (515 µL) were mixed in glass vials and heated at 95°C for ten minutes. The mixture was allowed to cool for 90 seconds before the addition of ammonium acetate buffer. The reaction was allowed to go to completion (10 minutes) and the absorbance of the mixture was recorded at 562 nm with a DR Lange, Cadas 30S spectrophotometer. To calculate the Fe concentration the absorbance of the mixture was compared to a five-point calibration curve made using a stock solution of FeCl_3_ (Merck pro analysis, 99% purity). A subset of samples was analyzed with ICP-AES and the comparison showed good correspondence between the two methods (Fe_ICP-AES_ = 1.04 Fe_Ferrozine_ – 0.22; r^2^ = 0.97, p<0.01, n = 7). The Fe transport capacity (α) of the river waters was estimated as the percentage of Fe that remained in solution at a salinity of 30 compared to the initial concentration at 0 salinity [16].

Organic carbon (OC) was analyzed by high temperature catalytic-oxidation in a Shimadzu TOC V-CPN analyzer. A four-point calibration curve was used in order to correct the results. Blanks and calibrated standards were included in each run.

Colored or chromophoric OM is the fraction of OM that absorbs UV and visible light and UV-Vis spectra can be used to extract structural information about the OM [34]. Absorbance was analyzed using a Beckman Coulter DU-800 spectrophotometer. The ratio of the absorbance measured at 250 and 365 nm (E_2_∶E_3_) was used as an estimate of the relative size of OM molecules [35]. High molecular weight OM has a stronger absorption at larger wavelengths. Thus, a higher E_2_∶E_3_ ratio corresponds to a lower molecular size of the organic matter, and the range of E_2_∶E_3_ values measured on *in situ* river waters (4.3–5.7) correspond to average molecular weights between 1100 and 4100 D [35]. The ratio of the absorbance measured at 465 and 665 nm (E_4_∶E_6_), on the other hand, is considered a to be a general tracer of humification (e.g., related to higher molecular size and aromaticity and lower O∶C and C∶N ratios) [34], [36]. While FA and humic acids (HA) are strictly only separated by their acid-base solubility characteristics (i.e. FA are soluble at any pH and HA precipitate below pH 2), FA are generally of lower molecular weight and aromaticity. FA and HA generally exhibit E_4_∶E_6_ ratios between 5–12 and 14–24, respectively [36]. Specific ultraviolet absorbance at 254 nm (SUVA_254_) is commonly assumed to be correlated with dissolved OM aromaticity, with values>3.5 corresponding to an aromaticity above 25% [37], and was calculated by dividing absorbance at 254 nm by the concentration of OC of the different rivers.

The Fluorescence Index (FI) is the ratio of emission intensities at 470 and 520 nm at an excitation of 370 nm and can be used as a reflection of the hydrophobicity of the OM [38], [39]. Fluorescence was measured with a Varian Cary Eclipse fluorospectrophotometer at a range of emissions going from 300 to 600 nm and collected over an excitation wavelength ranging from 250 to 500 nm. Excitation and emission slit width were set to 5 nm. The fluoro-spectrophotometer was zeroed with an empty quartz cuvette and a milli-Q water sample was run before every set of analysis to be used as a blank. FI was calculated according to [40] and by the resolution of the equations used in [41]:
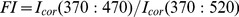
(1)


The intensities were corrected for inner-filter effects as:
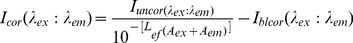
(2)


Where *I_uncor_*(λ_ex_: λ_em_) is the uncorrected intensity for each pair of excitation-emission wavelengths and L_ef_ is the effective path-length of the cell; the value of L_ef_ is estimated to be 0.5 cm for a 1 cm cuvette [41]. A_ex_ and A_em_ are the absorbance values at the excitation and emission wavelengths, respectively. I_blcor_(λ_ex_: λ_em_) is the intensity of fluorescence for the wavelength pair of interest for a milli-Q water blank.

### Statistical analysis

To test for long-term trends in Fe and OM concentrations, non-parametric Mann-Kendall tests for trends in time series were used. To estimate the magnitude of a trend that was significant by Mann-Kendall, we compared the median value during the first 5-year period (1972–1976) with that of the last 5-year period (2008–2012). Comparison of the relative magnitude of the trend was tested by paired t-tests. Co-variation between chemical characteristics among the sampled river waters was tested by Pearson correlations. Linear regressions were used to explore which variables may explain variation in the change in Fe∶OC and Fe transport capacity with increasing salinity. Assumptions of normality for paired t-test, Pearson correlations and residuals of linear regressions were verified by Kolmogorov-Smirnov tests. The dependence of Fe solubility on experimentally manipulated levels of pH was tested by non-parametric Spearman rank correlation.

Mann-Kendall tests were performed using an Excel macro, MULTMK/PARTMK, developed by Anders Grimvall and Claudia Libiseller, Linköping University, in collaboration with The Swedish University of Agricultural Sciences. All other statistical tests were performed in PASW Statistics 21.

## Results

### Chemical characteristics of the sample waters

**Figure 1:**
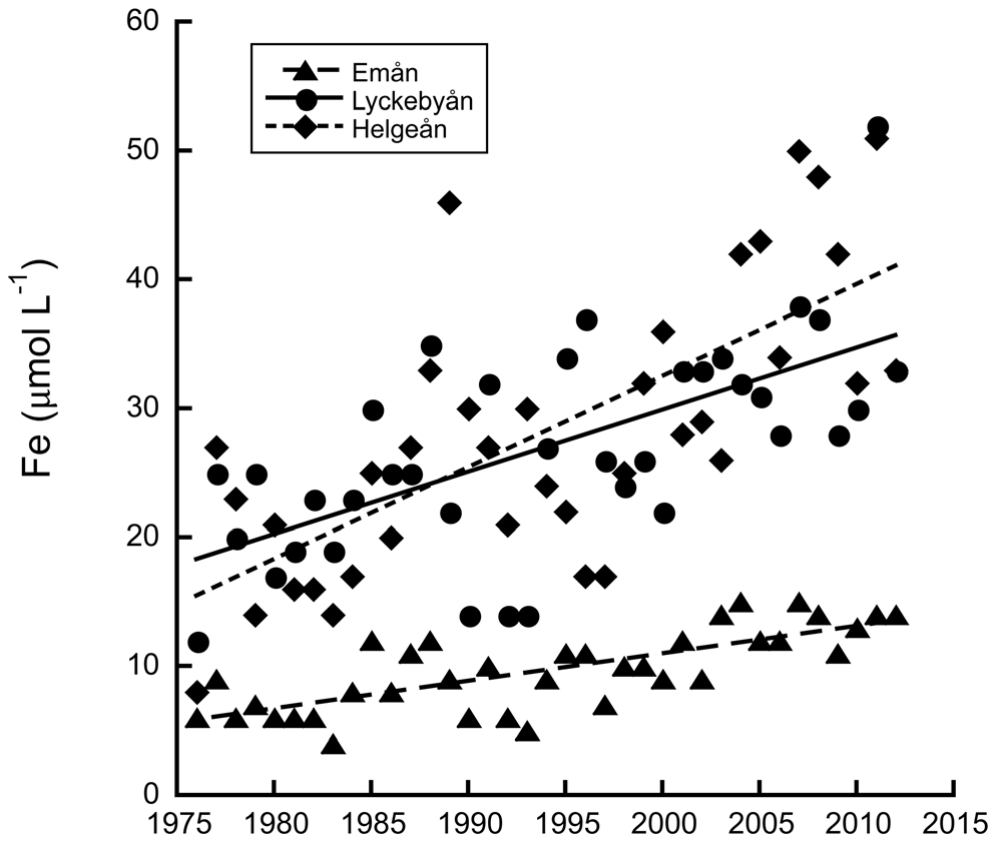
Yearly mean iron concentrations in the river mouths of three different rivers from 1976–2012. Lines denote the linear regression equations, which were µmol Fe L^−1^ = 0.212× year – 413.2 (r^2^ = 0.56, p<0.001); µmol Fe L^−1^ = 0.477× year – 924.0 (r^2^ = 0.40, p<0.001); and 0.716× year – 1398.4 (r^2^ = 0.51, p<0.001) for Emån, Lyckebyån and Helgeån respectively.

The rivers chosen for this study exhibit a strong and linear increase in Fe concentrations (Mann-Kendall p<0.001 for all rivers) as exemplified by Fig. 1. Median Fe concentrations were 7–25 µmol Fe L^−1^ higher for the period 2008–2012 than in the beginning of the monitoring series (1972–1976), corresponding to an average increase of 260% (Table 1). When comparing OM during the same period, there was also a significant increase (Mann-Kendall p<0.01 for all rivers) albeit a significantly smaller one (on average 82%, paired t-test p<0.05, d.f. = 6).

At the time of sampling the waters from the rivers encompassed a range of chemical characteristics (Table 2). The variation in OC was almost 2-fold and the variation in Fe concentration was 3.5-fold. Higher OC concentration did not always correspond to higher Fe concentration (r = 0.12, p = 0.76), and the molar Fe∶OC ratio was variable from 0.006 to 0.022. Analyses of absorption spectra revealed some variability in optical characteristics between the waters. The largest variation was seen in E_4_∶E_6_ ratios, reflecting a relative dominance of FA over HA in, e.g., Emån compared to Ljungbyån. While there was also some variability in E_2_∶E_3_ ratios—indicative of molecular size—SUVA_254_ and FI were remarkably invariable, which should reflect a similar degree of hydrophobicity of the OM in the waters. Among the water samples, there was a negative correlation between E_2_∶E_3_ ratios and Fe concentrations (r = −0.76, p<0.05) and between E_2_∶E_3_ ratios and Fe∶OC ratios (r = −0.68, p<0.05), indicating that waters with OM of a relatively larger molecular weight carries more Fe.

**Table 2:** Iron and organic matter concentrations and organic matter quality indicators of the experimental waters.

	sampling	pH	OC	Fe	Fe∶OC	E_2_∶E_3_	E_4_∶E_6_	SUVA_254_	FI
	date		(mmol L^−1^)	(µmol L^−1^)	molar ratio 10^−3^			(L mg^−1^ m^−1^)	
Emån I	Jul-12	6.9	1.6	10.2	7	5.1	7.8	4.6	1.38
Emån II	Nov-12	7.0	1.6	14.3	9	4.9	9.7	3.6	1.38
Ljungbyån	Dec-12	6.8	2.0	12.1	6	5.2	11.4	3.5	1.46
Lyckebyån	Jul-12	7.2	1.4	30.6	22	4.7	9.7	4.7	1.41
Mörrumsån	Dec-12	7.6	1.1	9.5	8	5.7	10.5	3.2	1.38
Helgeån I	Jun-12	7.8	1.1	17.3	16	5.1	10.4	4.6	1.43
Helgeån II	Nov-12	7.4	1.8	32.9	18	4.4	8.3	4.2	1.40
Lagan	Jan-13	7.2	1.2	17.9	15	4.6	7.8	4.1	1.39
Nissan	Jan-13	7.5	1.4	22.0	16	4.3	7.7	4.3	1.40

### Salinity gradients and Fe additions

**Figure 2:**
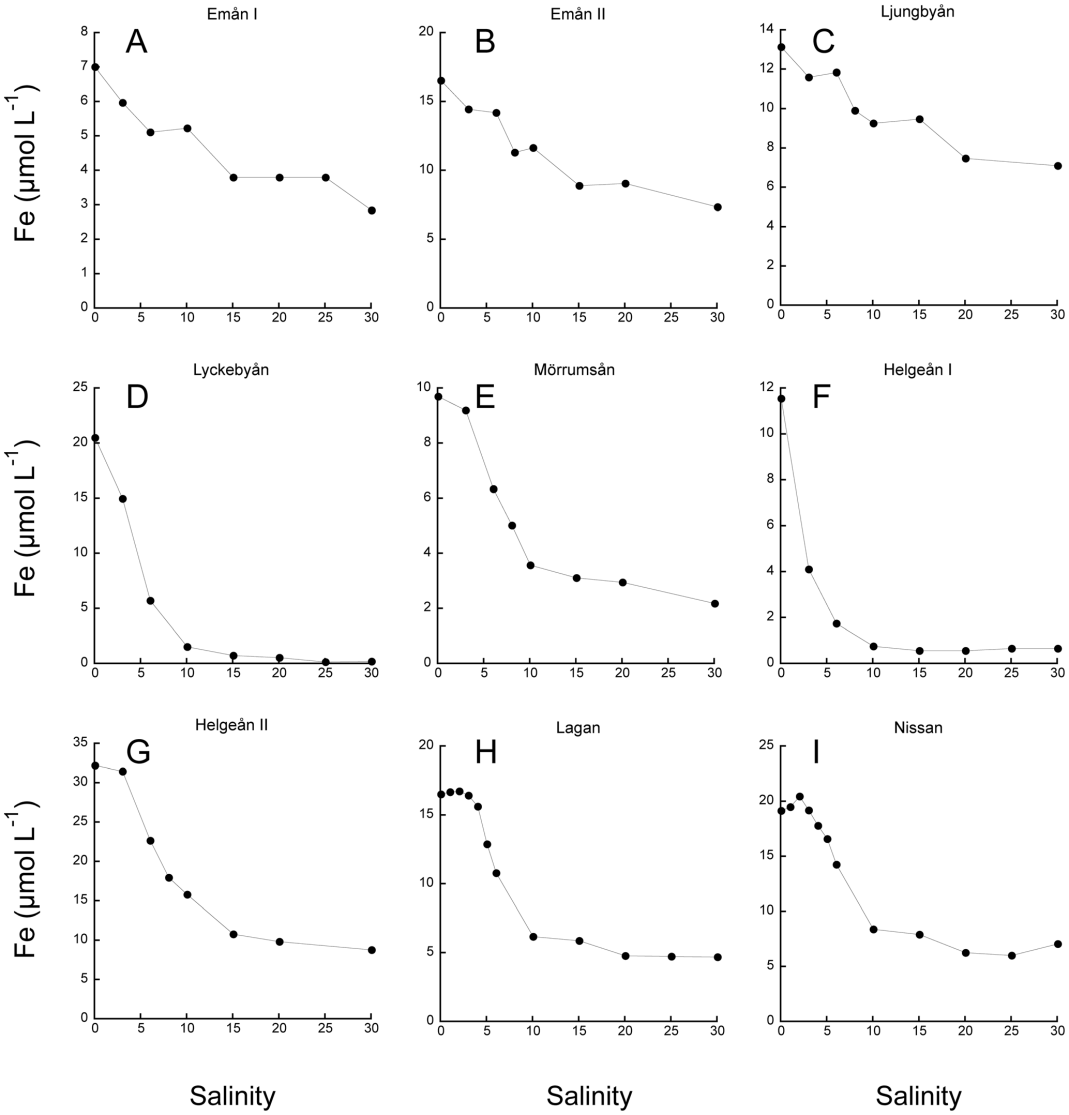
Concentration of iron in suspension at different salinity after addition of artificial sea salt.

In the experiments with artificial salinity gradients, there was a general pattern where Fe concentration in suspension decreased with salinity until approximately 15 where the Fe concentrations stabilized (Fig. 2). In waters from Lyckebyån and Helgeån I there was a sharp reduction in suspended Fe in response to increasing salinity (Fig. 2D and F), and the fraction remaining in suspension at high salinities (i.e. transport capacity, α) was 1 and 7%,respectively (Table 3). In the other waters the loss of Fe in suspension was less pronounced and 23 to 54% of the initial Fe remained in suspension at high salinity (Table 3). At a salinity of 30 the Fe maintained in suspension by the OM varied from 0.2 to 8.8 µmol Fe L^−1^. At a salinity of 6, which is typical for the Baltic Proper, 1.8 to 22.7 µmol Fe L^−1^ remained in suspension.

**Table 3:** Iron in suspension at different salinities and iron transport capacity (α) for the different waters.

	Starting from ambient Fe concentrations			After Fe additions	
	Fe at salinity 6	Fe at salinity 30	α	Fe at salinity 6	Fe at salinity 30
	(µmol L^−1^)	(µmol L^−1^)	(%)	(µmol L^−1^)	(µmol L^−1^)
Emån I	5.1	2.4	35	6.8	4.8
Emån II	14.2	7.7	45		
Ljungbyån	11.9	7.1	54		
Lyckebyån	5.8	0.2	1	3.8	0.4
Mörrumsån	6.4	2.2	23		
Helgeån I	1.8	0.7	7	0.2	0.2
HelgeånII	22.7	8.8	27		
Lagan	10.8	4.7	29		
Nissan	14.3	7.1	37		

The manipulation of pH resulted in a range of pH from 6.5 to 8.0, which encompassed the range in the samples of the experimental salinity gradients of all river waters (6.8–8.0). The effect of pH on aggregation was negligible, i.e. the coefficient of variation of Fe in suspension across the pH range was <5%, while it was 32–141% in the experimental salinity gradients, and there was no significant relationship between pH and Fe in suspension (p>0.58).

**Figure 3:**
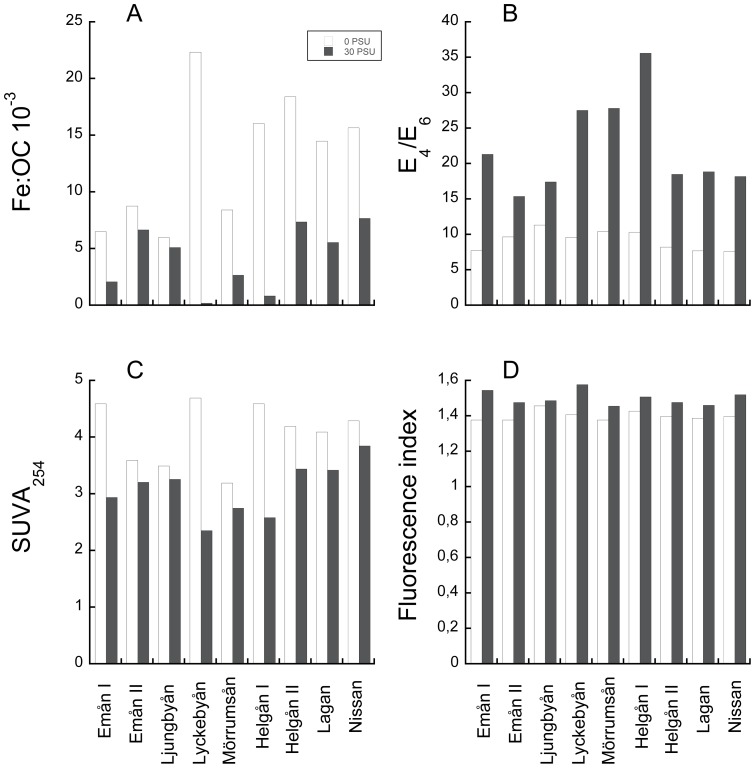
Differences in iron and organic matter in suspension in river waters at 0 and 30 salinity. A) ratio between iron and organic carbon, B) ratio of absorbance at 465 and 665 nm, C) specific UV absorbance at 254 nm and D) fluorescence index (ratio of emission at 470 and 520 nm and an excitation of 370 nm).

The extensive loss of Fe at increasing salinity was contrasted by quantitatively small losses of OM, which is reflected as a strong reduction of Fe∶OC ratios from 0 to 30 salinity (Fig. 3A, p<0.005). The higher the Fe∶OC ratio at 0 salinity, the larger was the reduction in Fe∶OC from 0 to 30 salinity (r^2^ = 0.82, p<0.001). For instance in waters from Lyckebån and Helgeån I, Fe∶OC decreased from very high to very low levels whereas in waters with low *in situ* Fe∶OC (Emån, Ljungyån, Mörrumsån) the decrease after salt addition was modest. While the quantitative loss of OC was small, clear differences in the quality of the organic matter were seen when comparing UV-Vis and fluorescence properties at 0 and 30 salinity (Fig. 3B–D). Significantly higher E_4_∶E_6_ ratios at 30 salinity suggest that HA were preferentially lost from suspension in relation to FA (Fig. 3B, p<0.001). Moreover, lower SUVA_254_ (Fig. 3C, p<0.01) and higher FI (Fig. 3D, p<0.001), are indicative of a loss of relatively more aromatic OM in saline conditions. Differences in E_2_∶E_3_ ratios did not vary consistently between 0 and 30 salinity (p = 0.27).

**Figure 4:**
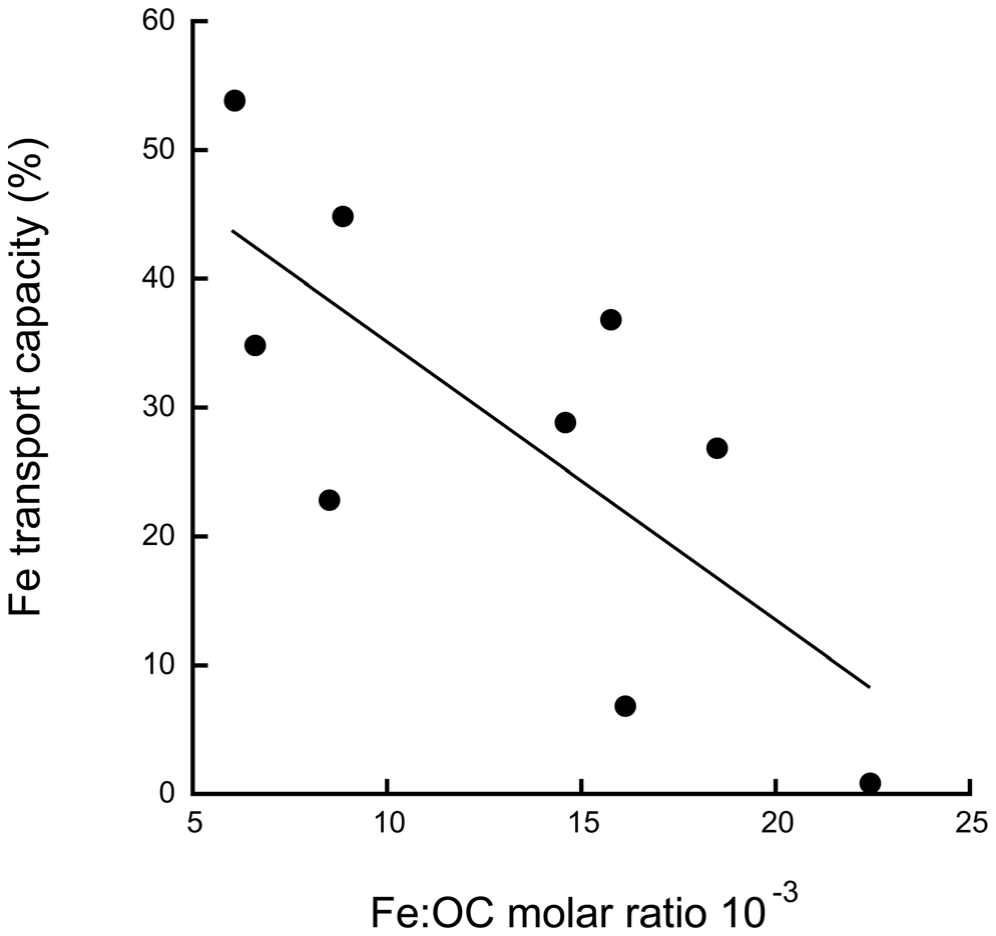
Relationship between the fraction of initial iron remaining in solution (iron transport capacity) at a salinity of 30 and the molar iron:organic carbon ratio of the river water (r^2^ = 0.54, p<0.05).

The ability of the different river waters to maintain Fe in suspension was best explained by the initial Fe∶OC ratio (Fig. 4, r^2^ = 0.54, p<0.05). The less Fe per organic carbon, the higher the transport capacity (α). Some of the variation in the relationship between Fe∶OC and α was explained by the E_2_∶E_3_ ratio at 0 salinity (r^2^ = 0.45, p<0.05) and 30 salinity (r^2^ = 0.87, p<0.001) respectively. Hence, the Fe transport capacity of the OM was negatively related to the Fe∶OC ratio and positively related to molecular size in the riverine water and the molecular size of the OM that remained in suspension at high salinity.

**Figure 5:**
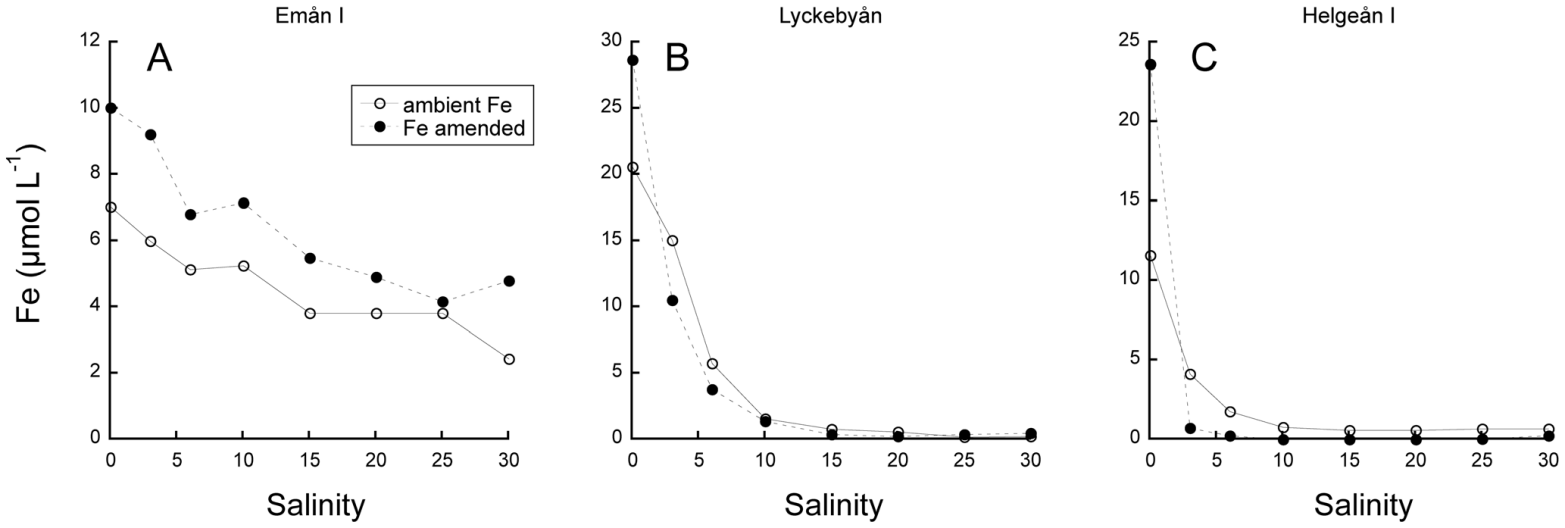
Concentration of iron in suspension at different salinity with and without iron amendments.

The addition of Fe was based on extrapolation of the linear increase in Fe over time, as displayed in Fig. 1, and corresponded to an increase from 10.2 to 15.6 µmol Fe L^−1^ for Emån, 30.6 to 46.5 µmol Fe L^−1^ for Lyckebyån and 17.3 to 30.5 µmol Fe L^−1^ for Helgeån. Increasing the initial Fe∶OC ratio by additions of Fe reduced the ability of the OM to maintain Fe in suspension in waters from Lyckebyån and Helgeån I, where Fe∶OC ratios were already high (Fig. 5B and C, Table 3). For these waters, the Fe additions resulted in lower concentrations of Fe in suspension than without Fe additions at low salinities, while they resulted in relatively lower or similar concentrations of Fe in suspension at high salinities. Fe∶OC at high salinities were little affected by Fe addition in water from Lyckebyån and Helgeån, suggesting that added Fe in these waters precipitated without interactions with OM. Contrasting to the behavior of these two waters, the water from Emån I, where Fe∶OC was initially low, maintained even some of the added Fe in suspension at high salinity (Fig. 5A, Table 3). While Fe concentration was enhanced, OC concentration was not affected by Fe addition, i.e. Fe∶OC was higher, suggesting that the ambient OM had the capacity to keep more Fe in suspension. At zero salinity, all waters had the capacity to keep additional Fe in suspension.

## Discussion

Little is known about how increases in riverine Fe concentrations, as reported here and elsewhere [12], [13], may affect the receiving estuarine and marine ecosystems. A key question is the extent to which the riverine Fe aggregates/sediments or remains in suspension as it mixes with seawater. The results obtained here suggest that the Fe transport capacity of the studied river waters is variable in space and time but generally high in light of the general concept of fast and efficient Fe removal during estuarine mixing. The amount of Fe in relation to OM, as well as the character of the riverine OM, appears to play a significant role to the Fe transport capacity of the studied river waters.

Loss of Fe from suspension through aggregation increased with salinity, which is consistent with earlier studies, e.g. [5], [6], [42]. Several mechanisms may cause the destabilization of Fe along salinity gradients. A major factor may be the neutralization of negatively charged functional groups of the OM by magnesium and calcium, resulting in co-precipitation of Fe and OM [5], [43]. Alternatively, hydrophobic Fe-OM may be “salted out” of the water phase due to reduced space between water molecules at higher salinity [44], [45]. Moreover, Fe-OM colloids may aggregate into larger particles in contact with seawater [17], [42] because of neutralization of surface charge that would otherwise separate colloids by electronic repulsion [46]. Additionally, Fe, magnesium, and calcium compete for binding sites of organic ligands [47], and the increasing concentrations of these competing cations in the marine water may result in the release and hydrolysis of Fe. While pH is known to strongly influence the speciation and solubility of Fe [31], [48], aggregation in response to pH differences alone was insignificant, suggesting that pH was of secondary importance to salinity in controlling aggregation of Fe. In two of the river waters (Helgeån I and Lyckebyån), only a few percent of the Fe remained in suspension at high salinity, which is in line with the generally low estimates of Fe transport capacity [15], [21]. However, in the seven remaining river waters, a considerably larger fraction of Fe (≥23%) remained in suspension at high salinities, which is closer to Fe transport capacities reported by, e.g., Krachler et al. [16], [17] for peat draining waters (∼22%) and Shiller and Boyle [49] for the Mississippi River (∼33%).

Overall, the Fe transport capacity of these waters ranged from 1 to 54%, and the higher the Fe∶OC ratio of the riverine water was, the more Fe aggregated. That a higher Fe∶OC ratio would make Fe more prone to aggregate may have several explanations. First, Fe is distributed between two main pools; complexes with organic macromolecules that are generally smaller than 10 kD or 0.5–4 nm, and Fe colloids (presumably Fe(oxy)hydroxides) that are generally larger than 10 kD or 3–50 nm and bind OM at the surface, which enhances colloidal stability [50]–[53]. Estuarine mixing experiments have indicated that the Fe-rich colloids from river water aggregate into larger particles in contact with seawater, while the organic rich phase is little affected [17], [42], [54], [55]. For instance, Pokrovsky et al. [54] reported a strongly non-conservative behavior of colloidal Fe and OM and that the size fraction below 1 kD was not affected during estuarine mixing of an Arctic river. Thus, the range of Fe aggregation in the different river waters, and its relation to Fe∶OC, may reflect the relative contribution of organic rich complexes and Fe-rich inorganic colloids respectively. Secondly, the larger the organic fraction of a colloid, the lower the wet density, e.g., organically dominated colloids in aqueous solution may have densities similar to water (∼1 g cm^−3^) while colloids with a large inorganic fraction may have densities up to ∼2.5 g cm^−3^ [51].

When Fe was added to water from river Emån, which had a low Fe∶OC ratio, the concentration of Fe in suspension was enhanced across the salinity gradient. Potentially, this reflects the formation of stable Fe-OM complexes by some of the added Fe. On the contrary, when Fe was added to the high Fe∶OC waters of Lyckebyån and Helgeån, aggregation of Fe occurred at lower salinity and the amount of Fe in suspension was not enhanced. This may reflect the formation of Fe(oxy)hydroxides, possibly due to a lower availability of free carboxyl or phenolic functional groups in the OM of these two rivers than in Emån, and that the resulting increase in Fe-rich colloids resulted in aggregation of these colloids at lower salinity. Interestingly, the Fe transport capacity of water from Helgeån differed strongly between summer (7%) and winter (27%) while Fe∶OC was in fact slightly higher in winter, underlining that there is greater complexity to the issue than the ratio between Fe and OM.

In addition to the Fe∶OC ratio, there were strong indications that the composition of the OM was influencing the ability to maintain Fe in suspension. The relative molecular size of the OM in the river water (as indicated by the E_2_∶E_3_ ratio), and also of the OM that remained in suspension in the saline treatments, was positively correlated to the amount of Fe that remained in suspension at high salinity. Jirsa et al. [18] found that large size fractions of OM seemed to be most important for chelation of Fe. This may reflect the abundance of humic substances, in particular FA, which have been suggested to have a high potential to carry trace metals in saline waters [4], [16], [17], [55]. Krachler et al. [16] ascribed a difference in Fe transport capacity between two riverine waters (7 vs. 22%) to the abundance of terrestrially derived FA. Later work from the same group suggest that in particular lignin catabolites are important Fe carriers resistant to salt-induced aggregation [55]. Compared to HA, FA have lower molecular weight and aromaticity which should make them less prone to aggregate, which is supported by the increasing E_4_∶E_6_ (FA to HA fraction) with higher salinity in the mixing experiments. The higher charge density of FA has been suggested as a reason for their relatively high capacity to retain Fe in solution at increasing salinity [16], [20]. Thus, while the solubility of Fe may depend on FA of high molecular weight, the largest and most hydrophobic OM fractions were lost along the salinity gradient. This is in agreement with a conservative behavior of dissolved OM in general but a distinctly non-conservative behavior of a rather limited fraction made up of HA [56].

Complexes between Fe and terrigenous OM are strong, and have been suggested to play a major role to Fe solubility in OM rich river plumes and coastal waters [4], [57], [58]. This is in accordance with the results of the present study, which further indicate that riverine OM may facilitate Fe transport to open waters. The Fe transport capacities for these river waters and thereby the Fe provided to marine waters (0.2–8.8 µmol L^−1^) contrasts strongly with the Fe concentrations supposedly provided by the “average world river” (40 nmol L^−1^; [21]). The relatively high stability of Fe in these boreal river waters may depend on the abundance of strong FA ligands. For the Baltic Sea, the main carrier phase for Fe has been found to be carbon-rich FA associated compounds, likely of riverine origin [20]. While the Baltic Sea is a stoichiometrically low nutrient-high Fe system [23], strong complexation reduces Fe bioavailability [57], [59]. The high Fe concentrations in the Baltic Sea (15–144 nmol L^−1^; Bothian Sea - Baltic proper; [20]) have been put forward as one reason explaining the success of nitrogen fixing cyanobacteria [22], [23], which have a Fe demand 4–6 times higher than other phytoplankton [60]. At the same time cyanobacterial bloom development and nitrogen fixation has been suggested to be limited by Fe bioavailability [24]–[27]. Several studies demonstrate that Fe associated with natural OM can be effectively accumulated by phytoplankton [22], [61], [62]. Organically complexed Fe may become bioavailable through photochemical reduction of Fe(III) to Fe(II) [23], through biological release of superoxide for extracellular reduction of Fe(III) [63], and by the production of siderophores [64]. Hence, several studies propose that riverine inputs may be important in providing biologically available Fe [57], [65], [66]. In all, the ability of the Fe-OM to remain in suspension along the salinity gradient in this study, suggest that the increasing riverine inputs may provide increasing concentrations of biologically available Fe to the Baltic Sea that may favor cyanobacterial activity.

Given the decisive role of Fe to the structure and biogeochemical cycling of aquatic ecosystems, more research is needed to further our understanding of the consequences as well as the underlying causes of the strongly increasing riverine Fe export. Since Fe solubility relies on interactions with OM, it has been suggested that reported increases in OM concentrations [67], [68] is a main driver of increasing Fe concentrations [14]. However, the increase in Fe concentrations is generally higher than that of OM concentrations [12], and experimental additions of Fe to natural waters from the region show that the OM present in the water has the capacity to maintain vastly higher concentrations of Fe in suspension, e.g. current study,1[2], [19]. Instead, extended periods and areas of reducing conditions in hydrologically connected soils, as a result of wetter and warmer catchments, have been suggested as a factor enhancing Fe export from the catchment [12], [13], [69]. Weyhenmeyer et al. [70] further proposed that increasing precipitation and shorter water retention times in lakes have changed Fe processing along the aquatic continuum, so that reduced sedimentation of Fe in lakes may also contribute to the higher Fe concentrations in river mouths. In all, this suggest that climate change may enhance the flux of iron from boreal rivers to the marine system.

To understand the consequences to the receiving system, a better comprehension of the interactions between Fe and OM and how it affects Fe transport capacity and Fe bioavailability is fundamental. The stability of Fe-OM along the salinity gradient should be affected by the form of the Fe, i.e. whether it is Fe(II), Fe(III) ions or Fe oxyhydroxides, as well as the molecular composition of the OM. Techniques such as extended X-ray absorption fine structure (EXAFS) and infrared (IR) spectroscopy, should allow the interactions between Fe and OM to be studied in depth [71]. While the present study is focusing on the potential transport of riverine Fe to open waters, higher Fe concentrations are also likely to affect sedimentation of OM and phosphorus and can potentially act as a carbon and phosphorus sink through long-term storage in sediments [72], [73]. Further research is needed to elucidate both causes and consequences of increasing Fe concentrations in discharge from boreal rivers.
